# Functional Brain States Measure Mentor-Trainee Trust during Robot-Assisted Surgery

**DOI:** 10.1038/s41598-018-22025-1

**Published:** 2018-02-26

**Authors:** Somayeh B. Shafiei, Ahmed Aly Hussein, Sarah Feldt Muldoon, Khurshid A. Guru

**Affiliations:** 1 Department of Mechanical and Aerospace Engineering, University at Buffalo, SUNY, Buffalo, NY 14260 USA; 2 Applied Technology Laboratory for Advanced Surgery (ATLAS), Roswell Park Cancer Institute, Buffalo, NY 14263 USA; 3 Department of Urology, Roswell Park Cancer Institute, Buffalo, NY 14263 USA; 4 Department of Urology, Cairo University, Cairo, Egypt; 5 Department of Mathematics, University at Buffalo, SUNY, Buffalo, NY 14260 USA

## Abstract

Mutual trust is important in surgical teams, especially in robot-assisted surgery (RAS) where interaction with robot-assisted interface increases the complexity of relationships within the surgical team. However, evaluation of trust between surgeons is challenging and generally based on subjective measures. Mentor-Trainee trust was defined as assessment of mentor on trainee’s performance quality and approving trainee’s ability to continue performing the surgery. Here, we proposed a novel method of objectively assessing mentor-trainee trust during RAS based on patterns of brain activity of surgical mentor observing trainees. We monitored the EEG activity of a mentor surgeon while he observed procedures performed by surgical trainees and quantified the mentor’s brain activity using functional and cognitive brain state features. We used methods from machine learning classification to identity key features that distinguish trustworthiness from concerning performances. Results showed that during simple surgical task, functional brain features are sufficient to classify trust. While, during more complex tasks, the addition of cognitive features could provide additional accuracy, but functional brain state features drive classification performance. These results indicate that functional brain network interactions hold information that may help objective trainee specific mentorship and aid in laying the foundation of automation in the human-robot shared control environment during RAS.

## Introduction

Robot-Assisted Surgery (RAS) has revolutionized the field of surgery by incorporating improved 3D visualization, 10× magnification, and endowrist technology allowing for six degree of wrist freedom in a miniaturized fashion^1,2^. In this environment, the surgeon operates remotely from the console by controlling robotic tools during surgery. Developing analytical methods to quantify surgical performance in real-time is of great importance, as real-time surgical mentorship can provide qualitative feedback during RAS^3,4^.

RAS requires a surgeon to not only master motor skills (human-machine interaction) in order to operate the robotic surgical system (console), but also to develop cognitive competence while operating remotely with no tactile feedback^5,6^. Therefore, surgical skill monitoring is an essential part of surgical training as well as procedure safety evaluation^5,7^. In the shared environment between a surgeon and robot, a mentor robotic surgeon monitors the performance of trainees^8^ and provides them with helpful assessment feedback^5,9^, and guidance^8,9^. Further, the expert surgeon must switch from surgical console or monitor and follow a trainee on a dual console.

In this shared environment, trust plays a key role and can lead to an open communication^10^ and cooperation^11,12^ leading to quality decision making^13^, safe risk-taking^14^, and satisfaction^15,16^. Hence, mutual trust between team members is required^17–19^, and is a fundamental factor in predicting the success or failure of the team, especially during high risk states^20,21^. While trust is difficult to define, Ring and van de Ven^22^ define it as “confidence in another’s goodwill”. In complicated teamwork environments such as RAS, many unpredictable complications and unforeseen events may occur during surgery. Trust is critical to help team members manage stressful situations by relying on their collaborative performance^23^.

Different approaches for trust evaluation have been proposed: affect and cognition-based^24^. Both are associated with performance in different ways^24^ and influence the psychological state of a team. Affect-based trust is related to the emotional understandings between team members^25^. The focus of affect-based trust is mostly psychological^26^ and therefore not the purpose of this study. However, cognition-based trust is related to performance and the understandings between team members engaged in it^26^ especially when evaluating competence^27^, responsibility^28^, reliability^25,29^, integrity, and dependability^26^.

Cognition-based trust has also been proposed to positively impact team performance^26^. Lack of mutual trust between team members^30^ results in anxiety, stress, and disappointment^31^. These effects can negatively influence performance, reduce cognition-based trust and subsequently, affect-based trust^25^.

We sought to model and investigate the level of cognition-based trust, which an expert robotic surgeon (with over 10000 hours of console time) has with regard to surgical training while performing Urethrovesical Anastomosis (UVA; a simple surgical procedure) during radial prostatectomy and Lymph Node Dissection (LND; a complex risky surgical procedure). We examined the brain activity of the expert surgeon utilizing electroencephalogram (EEG) as the expert surgeon observed the trainees perform the surgical steps. Surgical performance was additionally categorized as “trustworthy” or “concerning” based on validated NASA Task Load Index (NASA-TLX) scores and written feedback of the expert mentor surgeon.

Using both cognitive and functional brain state features, we used machine learning methods to objectively quantify the trust relationship between the mentor and trainee surgeons during RAS. We were able to extract key cognitive and functional features of brain activity which were capable to discriminate between surgical performances. During UVA, only functional brain state features were selected to discriminate between trustworthy and concerning performances. During more complex procedures- LND, both cognitive and functional measures of brain activity were selected to differentiate surgical performances, but functional features continued to drive performance classification. The experimental design includes controlled EEG data of mentor while he is engaged in observing trainees’ performances. EEG features such as ‘level of engagement’, that may be affected by other external effects rather that trust, were calculated and their effects were considered in classification analysis. Factors like frustration, level of task complexity and level of engagement are other possible factors, effective on EEG activity that were considered. This consideration is because all these effects are influential in the level of cognitive trust between mentor and trainee. Hence, the proposed mentor-trainee trust evaluation algorithm is objective. By objectively monitoring mentor-trainee trust during RAS mentorship, our findings will allow the development of protocols for measuring trust. The developed protocols can be employed during surgery to ensure safety, and may also aide in development of shared control and automation for the human-machine (robot) environment.

## Results

In order to quantify the brain state of the expert surgeon, we extracted *12 cognitive* and *21 functional* measures of one expert’s brain activity from EEG recordings as he observed three trainees’ performances during 87 UVA and 83 LND operations, as part of the “Mind Maps” program (Methods). These measures represent total brain activity calculated across six cognitive systems^32^**:** frontal (F), prefrontal (PF), temporal (T), central (C), occipital (O), and parietal (Pa). The 12 cognitive features included mental workload (MW), mental load (ML - calculated for each of the six cognitive systems), situation awareness (SA), engagement (E), blink rate (BR), asymmetry index (AI), and completion time (CT). The 21 functional state features were extracted by calculating the average phase synchronization, using equation (7), within (*strength*) and between (*communication*) the six cognitive systems. The functional features were calculated for each of four frequency bands: $\theta$(4–8 Hz), $\alpha$(8–12 Hz), $\beta$(12–35 Hz), and $\gamma$(35–60 Hz), resulting in a functional feature space of 84 dimensions. Combining the cognitive and functional features resulted in a final feature space (96 dimensions). Clustering and classification of brain activity was performed using this final feature space.

### Surgical Performance Categorization

In addition to recording single expert surgeon’s EEG activity during surgery, at the end of each procedure, the expert surgeon also completed a subjective assessment of the performance of the trainee using the validated NASA-TLX questionnaire^33,34^. Based on the subjective assessment, trainee performance could be separated into two groups:“trustworthy” and “concerning” performances. Performance level (PL) was extracted from the performance score (PS) of NASA-TLX, PL = 20-PS^34,35^. Surgical procedures with PL > 11 were categorized as ‘trustworthy’ and others were categorized as ‘concerning’. The threshold of ‘11’ was suggested by the mentor as trainees who got score of PL > 11 were qualified to continue supervised performance on the console.

### Functional brain measures for Urethrovesical Anastomosis (UVA)

**Figure 1:**
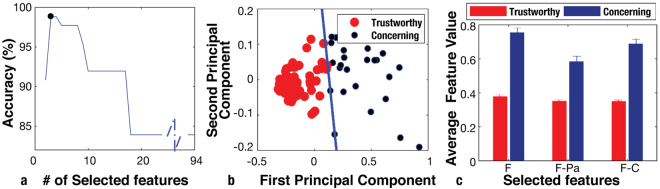
Selected features and classification results for UVA. **(a)** Classification accuracy by SVM method and LOOCV algorithm at different dimensions of selected features. Each training/testing sample used in LOOCV is the full 30 minute data from one surgery. **(b)** First and second principal components of features for UVA, classified using linear SVM method. **(c)** Average value and standard error of the mean (s.e.m) –standard deviation divided by the square root of number of data- of features for Trustworthy and Concerning procedures. Error bars represent s.e.m. for trustworthy (N = 63) and concerning (N = 24) samples. Selected features were significantly independent for trustworthy and concerning samples for selected features (two-sample t-test for 63 trustworthy and 24 concerning cases, resulted in P = 9.4 × 10^−25^, 1.03 × 10^−15^, and 4.9 × 10^−26^, respectively).

In order to determine which brain features were associated with trustworthy or concerning performances, we turned to a standard classification method from machine learning. Using support vector machine (SVM) classification^36^ with kernel target alignment (KTA)^37^, we derived a method for selecting the combination of features that provided the highest classification accuracy. These features are refered as *key features* (Methods). During UVA, the optimum number of discriminative features for accurate classification was three (Fig. 1a) with maximum accuracy of 98.81% calculated using the LOOCV algorithm. By using 10-fold cross-validation, the accuracy of trust evaluation for the UVA recordings was 95.40%, with F-score 94.25%. Use of 10-fold cross-validation decreased the classification performance, compared to resulted performance using LOOCV method. However, classification accuracy is still high, suggesting classification algorithm and selected features appropriate for classification of UVA recordings based on trustworthiness.

The results of the linear classification using LOOCV projected onto the first and second principal components seen in Fig. 1b. The accuracy of clustering the data set using 3 key features was additionally verified using fuzzy C-means clustering, resulting in an accuracy of 98.81% and J = 5.23. The two-sample t-test was applied to selected features to find the significance level of each feature difference in trustworthy and concerning performances.

### Cognitive and functional brain measures for Lymph Node Dissection (LND)

**Figure 2:**
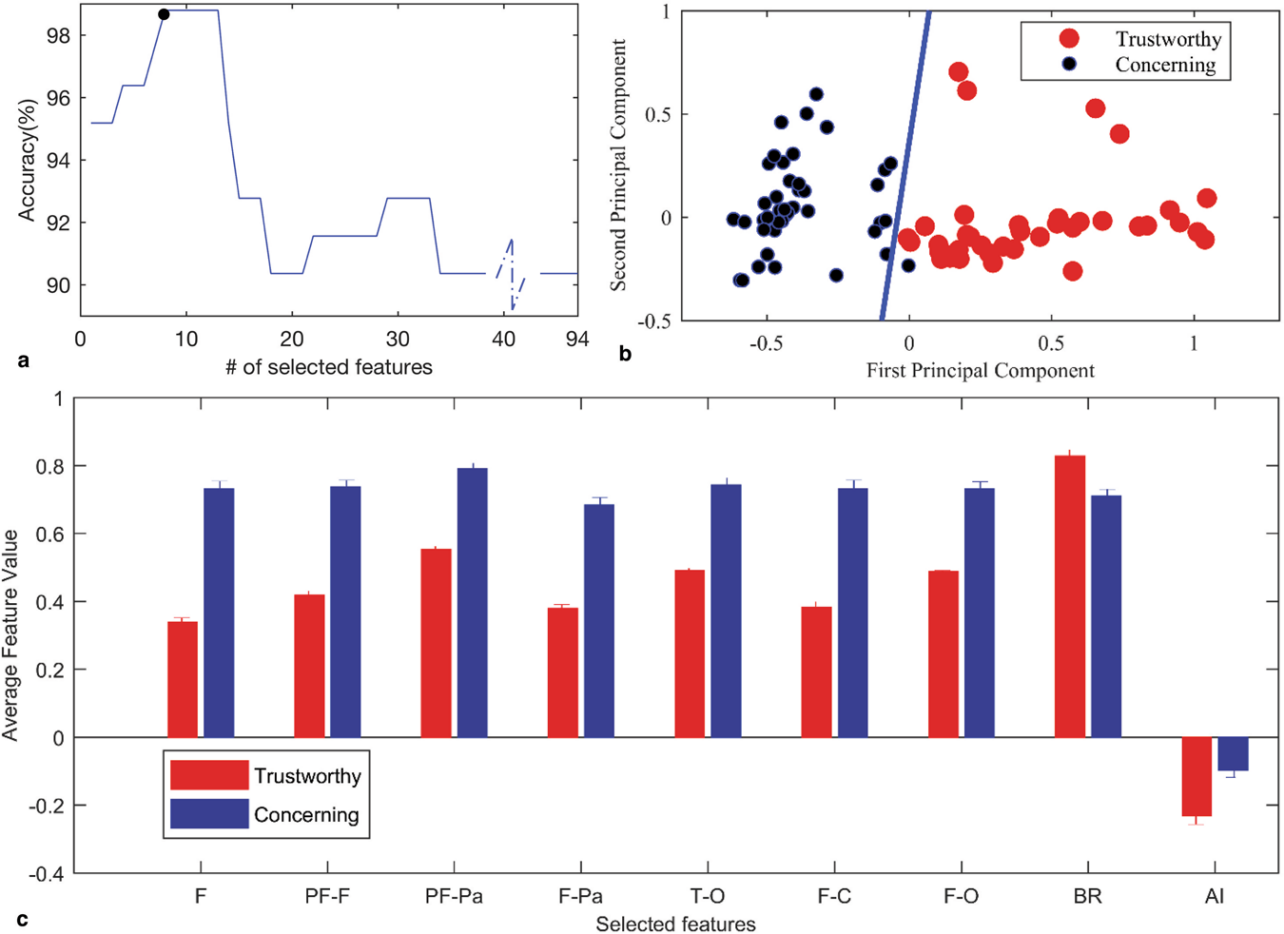
Selected features and classification results for LND. **(a)** Classification accuracy by SVM method and LOOCV algorithm at different dimensions of selected features. Each training/testing sample used in LOOCV is the full 30 minute data from one surgery. **(b)** First and second principal components of features for LND, classified using linear SVM method. **(c)** Average value and standard error of the mean (s.e.m) –standard deviation divided by the square root of number of data- significant selected features for Trustworthy (N = 43) and Concerning (N = 40) LND performances (all selected functional state features are from $\gamma$ frequency band). Two-sample t-test is used for evaluation, P = 1.2 × 10^−25^, 2.11 × 10^−23^, 4.6 × 10^−21^, 2.3 × 10^−21^, 8.1 × 10^−19^, 1.63 × 10^−19^, 1.5 × 10^−19^, 3.3 × 10^−5^, 1.7 × 10^−4^, respectively. Features ranked in descending order, as ‘F’ is better ranked than other featues and ‘AI’ has the lowest rank.

From a clinical point of view, LND is more complicated than UVAs as seen in our previous study^7^. This difference in complexity is also apparent in the classification results seen in this study. Although UVA could be categorized by using just three functional brain state features, more detailed features from both cognitive and functional feature sets were required for classifying LND. For these more complicated tasks, the incorporation of nine features resulted in the highest classification accuracy (using LOOCV method), 98.79% (Fig. 2a).

The projection of this classification onto the first and second principal components can be seen in Fig. 2b. Ten-fold cross-validation was also considered for classification. Accuracy of trust evaluation for the LND recordings was calculated as 93.97% with F-score 90.91%. Using 10-fold cross-validation, classification accuracy is decreased compared to the accuracy of classification of LND samples using LOOCV method. However, high classification accuracy even by using 10-fold cross validation, suggests that classification algorithm is reliable in discriminating trustworthy and concerning samples of LND recordings.The accuracy of clustering the data set using nine key features was additionally verified using fuzzy C-means clustering, resulting in an accuracy of 98.79% and J = 4.56.

**Figure 3:**
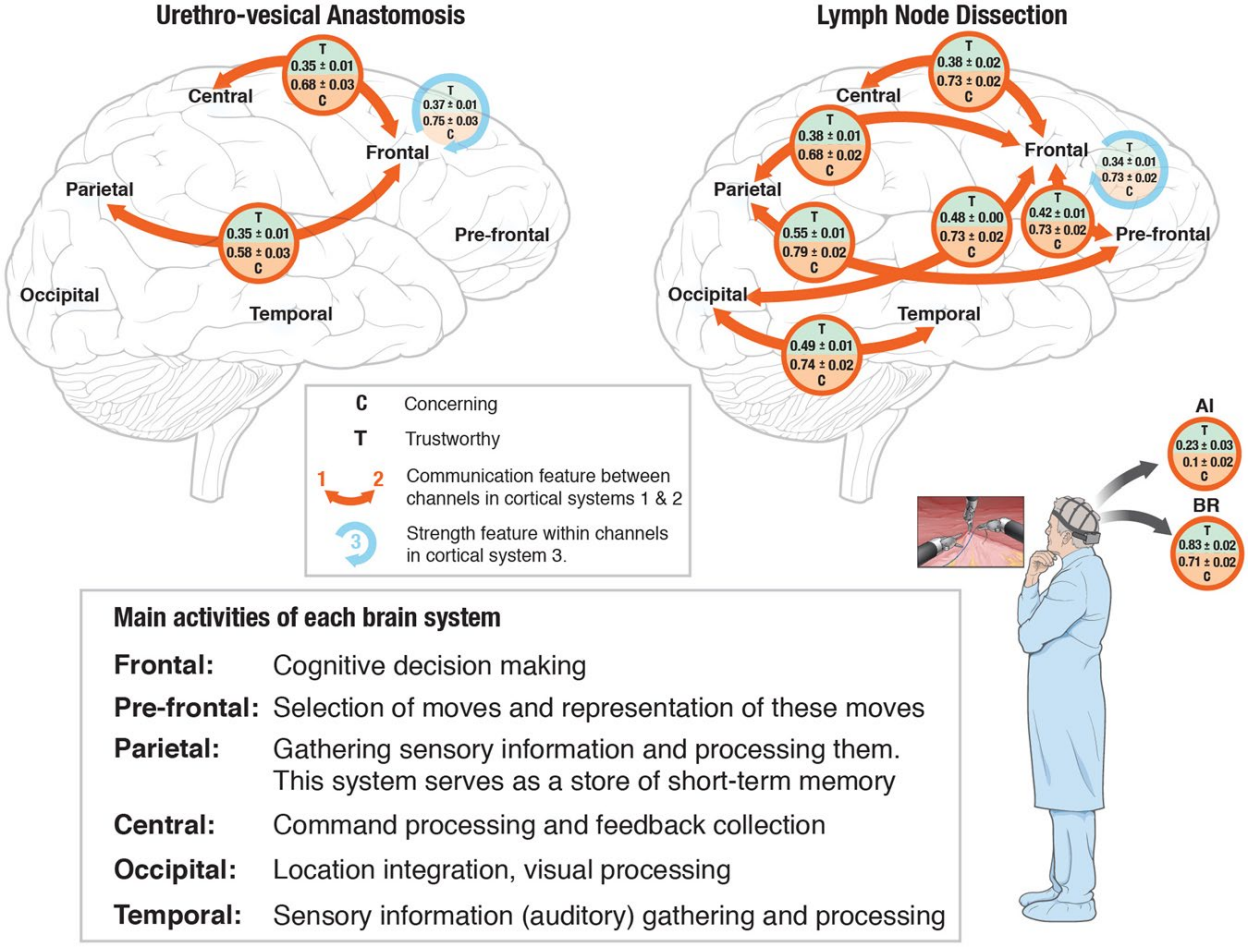
Illustration of discriminative brain features while observing trustworthy and concerning performances by robot-assisted surgical mentor. Functional connectivity features (strength and communication) for six systems of brain^32^ are shown for UVA and LND recordings. Values in orange circles represent the average and standard error of communication between channels in pairs of cortical systems for Trusthworthy (T) and Concerning (C) cases. Values in blue circles represent the average and standard error of strength within channels in the associated cortical system for Trusthworthy (T) and Concerning (C) cases. For UVA performances, functional connectivity features were able of discrimination. However, for LND performances, Asymmetry Index (AI) and Blink Rate (BR) features added discriminative information to functional state features to improve classification accuracy. Considering the distict function of each brain system, results can be interpreted as specific systems of the mentor’s brain (Frontal, Central, Parietal) being functionally connected in a different way for Trustworthy and Concerning cases. While for LND recordings, most functional connectivity features behave differently for Trustworthy and Concerning cases. Also, for LND recordings two cognitive features (AI and BR) showed different patterns for trustworthy and concerning cases.

Interestingly, the key features selected for this classification were drawn from both the functional and cognitive feature spaces and ranked by descending order shown in Fig. 2c and Table 1. While the top 7 key features used in the classification algorithm are again functional measures (Fig. 3) that largely involve the frontal cortex, the highest classification accuracy also required the incorporation of two cognitive features: blink rate (BR) and asymmetry index (AI).

**Table 1:** Stable significantly independent features, selected for classification of Trustworthy (N = 43) and Concerning (N = 40) LND procedures. A two-sample t-test is applied for significance evaluation. Feature values are represented as mean±standard error. ^****^And ^***^indicate correction multiplication test rejects null hypothesis and feature is significantly independent from other features with p-value ≤ 0.0001 and ≤0.001, respectively.

Feature Name	Trustworthy Feature Value	Concerning Feature Value	P-value
F strength^****^	0.34 ± 0.01	0.73 ± 0.02	1.2 × 10^−25^
PF-F communication^****^	0.42 ± 0.01	0.73 ± 0.02	2.1 × 10^−23^
PF-Pa communication^****^	0.55 ± 0.01	0.79 ± 0.02	2.3 × 10^−21^
F-Pa communication^****^	0.38 ± 0.01	0.68 ± 0.02	4.6 × 10^−21^
T-O communication^****^	0.49 ± 0.01	0.74 ± 0.02	8.1 × 10^−19^
F-C communication^****^	0.38 ± 0.02	0.73 ± 0.02	1.6 × 10^−19^
F-O communication^****^	0.48 ± 0.00	0.73 ± 0.02	1.5 × 10^−19^
BR^****^	0.83 ± 0.02	0.71 ± 0.02	3.3 × 10^−5^
AI^**^	−0.23 ± 0.03	−0.10 ± 0.02	1.7 × 10^−4^

For UVA, we found that the key discriminatory features were all functional measures of brain activity. As ranked by the classification method, the selected key features were *Frontal strength (F)*, *Frontal-Parietal communication (F-Pa)*, and *Frontal-Central communication (F-C)*. These features took on substantially different values between trustworthy and concerning procedures (Fig. 1c, Fig. 3, and Table 2). Notably, the frontal cortex was involved in all selected features for UVA recordings, as might be expected due to its role in conscious thoughts, decision making, cognition, and reasoning^38–41^.

**Table 2:** Stable significantly independent key features, selected for classification of Trustworthy (N = 63) and Concerning (N = 24) procedures for UVA (all selected features are from γ frequency band). A two-sample t-test is applied for significance evaluation. Feature values are represented as mean ± standard error- standard deviation divided by the square root of number of data. ^****^Indicates correction multiplication test rejects null hypothesis and feature is significantly independent from other features with p-value ≤ 0.0001.

Feature Name	Trustworthy Feature Value	Concerning Feature Value	P-value
F^****^	0.37 ± 0.01	0.75 ± 0.03	9.4 × 10^−25^
F-Pa^****^	0.35 ± 0.01	0.58 ± 0.03	1.03 × 10^−15^
F-C^****^	0.35 ± 0.01	0.68 ± 0.03	4.9 × 10^−26^

### Correlation of Engagement, Situation Awareness, and Mental Workload with key features

If two features in our feature space are significantly correlated, our feature selection method will only select one of these to be used by the classification algorithm. We examined the correlation between our selected key features and the remaining features. The correlation analyses helped to see if unselected features might also be useful for discriminating trustworthy and concerning procedures. These analyses also helped in interpretiton of the results. We found that engagement, and MW were significantly correlated with multiple key features for UVA (Table 3). Engagement, situation awareness, and MW were correlated with key features in LND (Table 4).

**Table 3:** Significant Pearson-correlations between key features, cognitive engagement level, situation awareness, and mental workload (MW) for LND (N = 83) procedures. Results are calculated for a 95% confidence interval.

Key Features	Non-key Features	Correlation	P-value
F	E	−0.56	1.0 × 10^−8^
F-Pa	E	−0.49	1.6 × 10^−6^
F-C	E	−0.46	6.8 × 10^−6^
F	MW	0.54	5.2 × 10^−8^
F-Pa	MW	0.53	9.5 × 10^−8^
F-C	MW	0.56	1.36 × 10^−8^

**Table 4:** Significant Pearson-correlations between key features, cognitive engagement level, situation awareness, and mental workload (MW) for LND (N = 83) procedures. Results are calculated for a 95% confidence interval.

Key Features	Non-key Features	Correlation	P-value
F	Engagement	−0.6	3.3 × 10^−8^
PF-F	Engagement	−0.49	2.6 × 10^−6^
PF-Pa	Engagement	−0.44	2.8 × 10^−5^
F-Pa	Engagement	−0.51	6.5 × 10^−7^
F-C	Engagement	−0.52	4.0 × 10^−7^
F-O	Engagement	−0.58	1.0 × 10^−8^
AI	Engagement	−0.30	5.0 × 10^−3^
T-O	Situation Awareness	0.33	3.0 × 10^−3^
F-C	Situation Awareness	0.25	2.0 × 10^−2^
F-O	Situation Awareness	0.41	1.0 × 10^−4^
AI	Situation Awareness	0.38	4.1 × 10^−4^
F	Situation Awareness	0.38	4.3 × 10^−4^
F-Pa	Situation Awareness	0.42	6.5 × 10^−5^
F	Mental Workload	0.45	1.6 × 10^−5^
PF-F	Mental Workload	0.30	0.006
PF-Pa	Mental Workload	0.34	0.001
F-Pa	Mental Workload	0.60	4.17 × 10^−8^
T-O	Mental Workload	0.43	4.43 × 10^−5^
F-C	Mental Workload	0.48	5.39 × 10^−6^
F-O	Mental Workload	0.44	2.81 × 10^−5^
BR	Mental Workload	−0.28	0.01

### Effect of the complexity of the task on results

LND and UVA are two distinct controlled tasks, for trust evaluation, that were considered in this study. Therefore, the analyses were applied to recordings from each task separately to take into account only the level of mentor surgeon’s trust on trainee’s performance. This statement was considered because the complexity level was consistent for recordings in each task – UVA and LND- separately.

However, to investigate the effect of task complexity level on classification results and find the driving factor (whether trustworthiness or complexity level), all LND and UVA recordings were considered together. To find if trustworthiness is driving factor or complexity level, we performed dependency statistical test and also classification analyses.

While the mentor was engaged in all recordings (trainee mentoring), he subjectively assessed the complexity of UVA and LND tasks as simple and complicated, respectively. Using two-sample t-test, we found that trustworthiness and complexity level are two significantly independent factors (*p-value* = *0*.*0089*).

We also classified all recordings (*combined data*: LND and UVA samples together). Here, we repeated classification algorithm for classifying *combined data* based on 1) *trustworthiness* (trustworthy and concerning groups) and 2) Their *complexity level* (complicated and simple groups). In all classifications (1 and 2), the same selected features (the same nine features selected in LND by KTA algorithm), LOOCV method, and the SVM classification method were used.

The accuracy of *complexity level* classification for *combined data* was *66*.*47%* (F-Score = *64*.*15%*). On the other hand, accuracy of *trustworthiness* classification for *combined data* was calculated as *95*.*29%* with F-score *94*.*11%*.

These results showed that the selected features are useful to evaluate *trustworthiness*, while *complexity level* may not be a driving factor toward the results of this article.

## Discussion

Currently, subjective assessment methods are common practical approaches in trust evaluation. Trust assessment using features extracted from brain activity can provide an objective method for addressing this challenge during robot-assisted surgery environment. Different types of trust in human-robot interaction environment, mostly human on human and human on autonomous robot, are challenging topics in this area^42,43^. Previously, robot trustworthiness and trust of human on robot has been studied^42,44,45^. However, focus of existing studies is mostly on the motion fluency of robots and trust of human on the autonomy of the robots^43^.

Previous work has explored the relationship between cognitive brain features and surgical performance^5,7^, correlations between cognitive features when performing vs. observing surgery^5,7^, or classification of satisfaction in brain computer interface environments^16^. However, these studies relied on calculations of cognitive features, some of which (MW, engagement) involve taking additional baseline information into account^46,47^.

The current study, first of its kind to our knowledge, evaluates the cognition trust of mentor on trainee performance (human-human). Trust was evaluated while trainee is performing very complicated hand motions to remotely control surgical robot tools (with no autonomy) during different surgical procedures in the operating room.

Here, we showed that by quantifying the mentor’s brain state we are able to achieve trust classification based on functional features alone for simple UVA procedures. Mentor’s brain state were quantified using functional brain networks and extracting simple measures of strength and communication between brain systems. For more complicated LND procedures, incorporating the Asymmetry index (AI) and Blink Rate (BR) into the functional feature space increased classification accuracy. However, these two cognitive measures are simple to calculate. These metrics were ranked in descending order (Fig. 2). We note that functional metrics drive the classification performance.

While functional features drive the classification of trustworthiness during RAS, we did observe a correlation between certain cognitive features and key functional features. Specifically, during UVA procedures, engagement and MW were found to be correlated with all three key functional features. As mentioned above, these two cognitive features require additional recordings of baseline EEG in their calculation^46,47^, making their calculation time consuming and potentially limiting their usefulness in real-time evaluation of trust during RAS. Given that the simpler measures of functional brain states provide similar information about the mental state during RAS, the functional features could be used to estimate MW. This application is useful, especially in clinical applications, where situational and other time concerns might impede performing more complex calculations.

During LND, we additionally observed a correlation between SA and multiple functional key features. SA is designed to measure the level of cognitive integration by assessing the difference in the PSD of the frontal region between the theta and gamma frequency bands. We observed a positive correlation between SA and multiple measures of communication (F-Pa, F-C, F-O, T-O; all extracted from the gamma band). This demonstrates a link between cognitive integration as assessed through frequency dependent activation of the frontal cortex and the co-activation of brain regions as assessed through measures of functional communication.

It should also be noted that we observed lower levels of functional strength and communication measures in trustworthy as compared to concerning procedures. This reflects lower levels of synchronization between brain regions, which may be an indication that more brain regions are invoked in the observation of concerning procedures. During LND, we also saw that the BR is lower during concerning procedures, potentially indicating increased concentration. The AI is also less negative during concerning LND, indicating higher levels of stress, fear, surprise, and possible disappointment.

These proposed metrics for objective trust evaluation were applied on mentorship data during RAS. However, this methodology can have many other applications in which cognitive trust plays an influential role, and will be essential in assessing and evaluating training of surgeons in RAS.

EEG features have been previously used by ET Esfahani *et al*.^48^ to evaluate the level of human satisfaction in human-humanoid robot interaction. They used cognitive EEG features of power spectral density and the Lyapunov Exponent measures to classify level of human satisfaction of robot motion direction into three categories of neutral, satisfied and not satisfied groups. They could find classification rate of 79.2%. They also investigated the dependency of their result on subjects. They reported expectation of higher accuracy (80.2–94.7%) for subject based emotional classification. Although the current study evaluates trust between two humans (mentor and trainee) and the methodology proposed in this study is different from the one used by Esfahani *et al*., we compared our results with the study by ET Esfahani *et al*.^48^ to find advantages and shortcomings of our proposed features in evaluating emotional states of trust using EEG data. EEG data used for extracting features in this study include 30 minutes recordings from mentor surgeon’s brain during supervising surgical tasks using 20-channel EEG headset, compared to 1–2 second recordings from subject’s brain during emotional response to the motion of humanoid robot using 14-channel EEG headset in Esfahani’s study. Our results show a high accuracy of 95.29% in classifying EEG recordings of the mentor’s brain into two categories of trustworthy and concerning. Finding high accuracy, compared to the result of study by ET Esfahani *et al*.^48^, shows that functional connectivity features may be more informative in evaluation of emotions like trust in collaborative environments. However, this high accuracy may be affected by involving more than one mentors in the study. The Lyapunov Exponent measures also seem informative in evaluation of emotions like satisfaction level in human robot interaction as these features measure the sensitivity of a dynamical system to initial conditions^48^.

The purpose of our next study is to repeat the current study using more than one mentor and also add Lyapunov Exponent measures to our feature set to develope a more objective trust evaluation algorithm to be used in human-robot interaction environment. Our expectation is using the brain functional connectivity features proposed in the current study combined with Lyapunov Exponent measures proposed by ET Esfahani *et al*.^48^, consider most important aspects of brain map during functioning in human-robot interactions, and may result in more valid results in emotion evaluation.

## Methods

The “Mind Maps” program was initiated in 2013 to record EEG data of surgeons during RAS. All participants provided an informed consent to participate. The brain activity of an expert robotic surgeon was recorded while observing the operations of three trainees. Data in this study included 83 LND (two trainees performed 28 LNDs and one trainee performed 27 LNDs) and 87 UVA (each trainee performed 29 UVAs) during Cystectomy and prostatectomy procedures using the da Vinci surgical system. The study was conducted in accordance with relevant guidelines and regulations, and were approved by Roswell Park Cancer Institute Institutional Review Board (IRB: I-241913). On average recorded procedures took approximately 4 hours and trainees performed 30 ± 12 minutes on the UVA and/or LND. During this research study certain portions were considered, especially EEG portions in which the mentor observed trainee’s performances. Hence, the term ‘recordings’ throughout this study means EEG data recorded from one mentor surgeon while supervising a trainee – with each recording being approximately 30 minutes.

A 24-channel wireless electroencephalogram (EEG) recording device was used to monitor one mentor’s brain activity during all surgical procedures using an ABM X-24 neuro-headset (Advanced Brain Monitoring, Inc. Carlsberg, CA). Sensors were placed over frontal (F), temporal (T), parietal (Pa), central (C), and occipital (O) cortices. EEG data from each channel was sampled at 256 samples per second.

NASA-TLX is a gold standard and subjective measure of performance at various human-machine interface systems^33,34^. During a multi-dimensional rating procedure, scores are assigned to six indexes^33,34^:

- Mental Demand: Evaluates the level of mental/perceptual activity demanded to complete the task.
- Physical Demand: Level of physical activity required to complete the task.
- Temporal Demand: Level of time pressure the subject feels during completing the task.
- Performance: Quality level of outcome and the level of satisfaction of doing the task.
- Effort: evaluates how hard (mentally and physically) should the subject work to complete the task.
- Frustration: Level of negative (compared to positive) psychological emotions the subject feels while completing the task.

The score given by mentor surgeon to mental demand index was used to evaluate level of complexity of LND and UVA recordings. The scores given by mentor surgeon to overall performance index were used to categorize recordings into trustworthy and concerning groups (labeling).

### Data Pre-Processing

The recorded EEG data were raw signals contaminated with different types of artifacts such as eye-blink, muscle activity, and environmental effects. The algorithm proposed by Berka *et al*. and implemented in Advanced Brain Monitoring framework^46^ was used to detect these artifacts and decontaminate EEG data. EEG signal in the time domain, which includes 3, 5, or 7 data point spikes with amplitudes greater than 40 mV, were detected as saturation and excursions artifacts^46^. These artifacts were discarded from data^46^. Environmental artifacts were removed by applying a 60 Hz notch filter to EEG data^46,47^. The EEG data from channels were filtered with a band-pass filter (0.5–128 Hz)^46^. Artifacts including muscle activity and eye movement were detected using wavelet transform and discriminant function analyses (DFA) applied to the raw data^46,47^. Linear Discriminant Function Analysis (DFA) was applied to EEG data to detect data points contaminated with eye blink^46^. This DFA uses absolute value of the 0–2, 2–4, 4–8, 8–16, and 16–32 Hz wavelet coefficients from the 50th, 40th 30th, 20th, and 10th data points before and after the target data point from FzPOz and CzPOz as features to classify each data point into categories of eye blink, theta wave, or non-eye blink. Database available from Advanced Brain Monitoring framework, including selected data from healthy, sleep-deprived subjects, were used to train the DFA. EEG data contaminated with eye-blink were removed from next analyses^46^.

Decontaminated EEG data recorded from the mentor’s brain while observing each trainee performing specific surgery was considered in the analyses. Short Fast Fourier Transform (SFFT) with a one second Kaiser moving window was used to calculate the power spectral density (PSD) of EEG signal. A 50% overlap was considered for Kaiser moving window. Considering the whole data in each recording and each channel, data points larger than 3 times the standard deviation were marked as outliers^49^ and discarded from the data set (recordings).

### Parcellation of the brain into cognitive systems

Perception, action, and cognition tasks were processed by specific brain systems^32^. Based on the function of different cortices of the brain, six main sub-networks^32^ were considered here as active systems while processing RAS operations. These systems are *Frontal* (F; cognition and action; F3, Fz, F4, F7, F8 electrode channels), *Prefrontal* (PF; cognition; Fp1, Fp2 electrode channels), *Central* (C; action; C3, Cz, C4 electrode channels), *Temporal* (T; perception; T3, T4, T5, T6 electrode channels), *Parietal* (Pa; cognition; P3, Pz, P4, POz electrode channels), and *Occipital* (O; perception; O1, O2 electrode channels)^32^.

### Measurement of cognitive features

Cognitive features extracted by analyzing strength of brain activity were used in human-computer-interaction applications to find the cognition status of user’s brain^50,51^. We analyzed the following cognitive features to evaluate cognitive trust in human-RAS shared environment: mental workload (MW), mental load (ML), situation awareness (SA), engagement (E), blink rate (BR), asymmetry index (AI), and completion time (CT). Explanation of these features were summarized in Table 5.

**Table 5:** Cognitive and functional brain state features.

Feature	Description	Main extraction method
Mental Workload (MW)	Level of working memory during problem solving and analytical reasoning	Mental Workload Classifier: Linear DFA is used to extract classes of low and high mental workloads
Mental Load (ML)	EEG channel amplitude	Power Spectral Density -PSD analysis
Situation Awareness (SA)	Expertise in predicting risks and making appropriate decisions	Power Spectral Density-PSD analysis
Engagement (E)	Level of information-gathering, visual processing, and allocation of attention	Engagement Classifier: PSD bands are used as inputs, and logistic discriminant function analysis (DFA) is applied to find the level of engagement
Blink rate (BR)	Portion of signal data points contaminated with eye-blink	Linear Discriminant Analyses
Asymmetry index (AI)	Difference of power decreased in alpha at right and left frontal hemispheres	Power Spectral Density-PSD analysis
Completion time (CT)	Total time of performance	Difference between end and start of performance
Strength	Level of total functional connectivity within channels in a specific subsystem	Functional connectivity network (pairwise phase synchronization)
Communication	Level of total functional connectivity between channels from different subsystems	Functional connectivity network (pairwise phase synchronization)

### Mental Workload

To calculate MW, we used the framework developed by the B-Alert EEG series from Advanced Brain Monitoring (ABM) company, which has been frequently validated in different studies^46,47,52^. Briefly, this framework calculates a baseline value of the absolute and relative power spectral variables from the C3-C4, Cz-PO, F3-Cz, Fz-C3, and Fz-PO channels during mental arithmetic, grid location, and digit-span baseline tasks. These baselines have been recorded from 80 healthy subjects, and are available from ABM software. A two-class quadratic logistic discriminant function analysis (DFA)^46^ was used to extract the probability of presenting a high mental workload. The quadratic logistic DFA was established once for one mentor based on baseline data collected before surgeries. The main assumption in MW interpretation is that each person has a relatively fixed cognitive capacity^46^. Commonly, MW refers to the portion of a person’s total mental capacity which is loaded^53^.

### Mental Load

The ML for each brain system during each recording was defined in equation (1) as the total power amplitude ($A$) of channels in each of the six considered cortices during each recording (one mentor observed trainee performing surgery):

1 $$M{L}_{i}=\sum _{j}A(j),\,\,\,\,\,\,\,i\in F,\,\,PF,\,\,C,\,\,O,\,\,Pa,\,\,T;\,\,\,j\in i$$

### Situation Awareness

The awareness of environmental elements, anticipating their status in near future, and managing probable risks and emergency response^54^ helps surgeons overcome uncertain and stressful environments^55^. Therefore, one can consider three levels of SA^56^; perception of the data and environmental element (Level 1), cognitive integration to comprehend the current situation (Level 2), and projection of future states and events (Level 3). Here, we were interested in measuring SA at level 2, because EEG analysis has shown that SA at level 2 (cognitive integration) is associated with the higher activity in the theta (4–8 Hz), and the gamma (35–60 Hz) frequency bands in the frontal cortex (^F^)^49^. We therefore defined the level of situation awareness as equation (2):

2 $$SA=PS{D}^{F}(\theta )+PS{D}^{F}(\gamma )$$

### Engagement

Engagement reflects the spatial recruitment of the brain regions in processing tasks associated with decision making. These tasks include, but are not limited to, information gathering, visual scanning, audio processing, and attention concentration on one aspect of the environment while ignoring other distractions^46,47^. As with the calculation of MW, we used the framework developed by the B-Alert EEG series from Advanced Brain Monitoring (ABM) company. However, in this case, the baselines were drawn from 5 minutes of three different tasks (3-choice vigilance task, eyes open, and eyes closed).These baselines were recorded from one mentor at the beginning of the whole research study. Here, the absolute and relative PSD of the Fz-POz and Cz-POz channels were used in a four-class quadratic logistic discriminant function analysis (DFA) which returned an estimation of the engagement level^46,47^. The range of this estimation is between 0–1 with 0 being no engagement and 1 fully engaged.

### Blink Rate

Signal data points, in each recording while one mentor observed trainee performing, contaminated with eye blinks are detected and decontaminated during data preprocessing as described in ‘Methods’. The number of contaminated points (${N}_{c}$) in the signal divided by the total points in the signal ($N$) is defined as the blink rate in equation (3):

3 $$BR=\frac{{N}_{c}}{N}$$

### Asymmetry index: a representation of surprise and fear

Negative emotions such as surprise, frustration, fear, and concern have opposing effects on the activity of the right and left lobes of the frontal cortex^57^. Asymmetry index is defined as the difference between the power density decrease in the left and right frontal hemispheres in the alpha band^58^, normalized as equation (4)^58,59^. AI was obtained from each recording (one mentor observed a specific trainee performing about 30 minutes).

4 $$\begin{array}{c}AI=\frac{L-R}{L+R}\\ L=PS{D}_{\max }^{F3}(\alpha )-PS{D}_{\min }^{F3}(\alpha )+PS{D}_{\max }^{F7}(\alpha )-PS{D}_{\min }^{F7}(\alpha )\\ R=PS{D}_{\max }^{F4}(\alpha )-PS{D}_{\min }^{F4}(\alpha )+PS{D}_{\max }^{F8}(\alpha )-PS{D}_{\min }^{F8}(\alpha )\end{array}$$

The AI was calculated as the average value over the following pairs of electrodes: (F3 and F4), and (F7 and F8). During negative emotional stimulations, the right frontal lobe shows more intense activity (associated with lower $\alpha$ power^59^) compared to the left lobe^59–62^ (alpha power is inversely related to activation^58^).

### Completion Time

By synchronizing the recorded EEG and the associated video of the surgery, completion time was defined as the total time a trainee was performing a surgery. The completion time can be defined using the number of total data points in signal ($N$) and the data recording sampling frequency (${f}_{s}$) in equation (5).

5 $$CT=\frac{N}{{f}_{s}}$$

### Extraction of functional brain networks

There are several methods for mapping time series into a complex network^63^. Here, Phase Locking Value (PLV) was used to map EEG time series into a complex network of brain functional connectivity. PLV was used because our purpose was to analyze brain functional connectivity when information is transformed throughout areas of the brain. Information transformation occurs whenever two areas are phase-synchronized (locked). PLV was calculated by applying continuous Huang Transform (HHT)^64^ to EEG recordings.

We calculated the pairwise phase synchronization of electrode channels to analyze the functional connectivity of the brain across four different frequency bands of $\theta$, $\alpha$, $\beta$, and $\gamma$.

To find the phase ($\phi$) of the EEG signals, we applied the continuous HHT^64^ to decontaminated EEG recordings. The instantaneous phase difference at time $t$, (${\rm{\Delta }}{\varphi }_{xy}(t)$), for pair of channels ($x$ and $y$) can be calculated based on equation (6)^65^.

6 $${\rm{\Delta }}{\varphi }_{xy}(t)=|{\varphi }_{x}(t)-{\varphi }_{y}(t)|$$

Transferring the range of phase into the boundary ${\varphi }_{x}\in [-\pi ,\pi ]$, the phase difference for all pairs of channels was normalized by using the range of phase difference (${\rm{\Delta }}{\varphi }_{xy}^{\max }=2\pi$ and ${\rm{\Delta }}{\varphi }_{xy}^{\min }=0$).

The average phase synchronization index ${{\rm{\Gamma }}}_{xy}(FB)$ can be defined by using the equation (7)^66^:

7 $${{\rm{\Gamma }}}_{xy}(FB)=\frac{\sqrt{[{\sum }_{t}\cos ({\rm{\Delta }}{\varphi }_{x,y}^{FB}(t)){]}^{2}+[{\sum }_{t}\sin ({\rm{\Delta }}{\varphi }_{x,y}^{FB}(t)){]}^{2}}}{P}$$

where, $P$ is the number of data-points in the time series used for averaging, and $FB$ is the frequency band. Calculating ${\rm{\Gamma }}$ for all pairs of channels resulted in the creation of four independent, frequency based functional connectivity matrices^66^. The extracted matrices were then used to calculate the functional features used in surgical performance classification.

### Measurement of functional features

In order to assess functional brain features, we measured the functional connectivity of the brain by assessing the pairwise phase synchronization between electrodes as described above. We then quantified functional brain activity across the 6 defined cognitive systems by defining two measures of cognitive system functioning: strength and communication. These features are explained in Table 5.

### Strength

The strength of a cognitive system was defined as the average functional connectivity of electrodes within the system.

### Communication

Communication, $C$, between two cognitive systems ${k}_{1}$ and ${k}_{2}$, was defined as the average functional connectivity in electrode pairs where one electrode lies within the first system and the second electrode lies within the second system:

8 $${C}_{{k}_{1},{k}_{2}}=\frac{{\sum }_{i\in {k}_{1},j\in {k}_{2}}{{\rm{\Gamma }}}_{ij}}{(|{S}_{{k}_{1}}||{S}_{{k}_{2}}|)}$$

Where $|{S}_{k}|$ is the number of nodes in the cognitive system $k$, where $k=\mathrm{1...6}$, and ${k}_{1}\ne {k}_{2}$. Note that the strength of each cognitive system can be calculated by letting ${k}_{1}={k}_{2}$ in equation (8).

### Accuracy of clustering

The accuracy of clustering data into two categories of trustworthy and concerning was evaluated using fuzzy C-means clustering. In addition to a high accuracy of clustering, which was calculated by comparing the cluster label and real label for the data, the clustering cost criterion parameter ($J$), also should be high for good clustering^36^. $J$, defined in equation (9), represents the ability of method to separate two groups of data by maximum distance between cluster center points^36^:

9 $$J=tr({S}_{W}^{-1}{S}_{B})$$

where, ${S}_{B}$, the between-cluster scatter, and ${S}_{W}$ is the within-cluster scatter matrix.

### Classification of data and selection of key features

To classify data, we used a linear SVM in combination with kernel-target alignment (KTA) and kernel class separability (KTS criteria)^37^. This approach iteratively calculates kernel alignment with different weights for combinations of features. The numerical iteration continues until the convergence of the kernel. Feature weights which result in maximum KTA were selected as the output of the algorithm and ranked by descending order^37^. It was assumed that features with higher weights are more important features^67^. Key features were chosen to be the minimal set of highest ranking features, which result in maximum clustering accuracy^68^. The only parameter the KTA method requires is the number of features to be selected. To maximize the performance of feature selection using this method, this parameter was selected from 2 to the size of the feature set with an increment of 1. Finally, the number of features that resulted in highest classification accuracy was considered for data analysis.

The LOOCV and 10-fold cross-validation were used during trustworthiness classification. We used both LOOCV and 10-fold cross-validation methods to investigate the effect of the training dataset size on the classification performance^36^^,^^69^.
